# Development and validation of an enzyme-linked immunosorbent assay for antibodies against *Mycobacterium bovis *in european wild boar

**DOI:** 10.1186/1746-6148-4-43

**Published:** 2008-11-01

**Authors:** Olaia Aurtenetxe, Marta Barral, Joaquín Vicente, José de la Fuente, Christian Gortázar, Ramón A Juste

**Affiliations:** 1 Instituto Vasco de Investigación y Desarrollo Agrario (NEIKER-TECNALIA), Berreaga 1, 48160 Derio, Spain; 2 IREC (CSIC-UCLM-JCCM), Ronda de Toledo s/n, 13071 Ciudad Real, Spain

## Abstract

**Background:**

Bovine tuberculosis (bTB) remains a significant problem in some parts of Spain largely because of contacts between cattle and wildlife reservoirs in extensive grazing systems. European Wild boar (*Sus scrofa*) is one of the species involved in the transmission of the disease to other species. Fast and simple detection methods would be critical for assessing infection prevalence, study the mechanisms of pathogen transmission and monitoring the effects of TB control measures.

**Results:**

An enzyme-linked immunosorbent assay (ELISA) to detect antibodies against *Mycobacterium bovis *in wild boar serum was developed and validated on 185 sera from TB positive and negative wild boar. Based on antigen inoculation of captive animals as well as tuberculosis compatible lesions, culture results and molecular analysis of hunted individuals, animals were allocated into two groups: tuberculosis positive group and tuberculosis negative group. After optimization of the positive to negative ratio using different combinations of serum dilutions and conjugate concentrations, the test yielded a sensitivity of 72.60% and a specificity of 96.43% for the best cut-off.

**Conclusion:**

Although some negative group animals showed an ELISA positive reaction (< 3%), this assay showed a high potential for accurate diagnosis of TB in wild boar, as its large dynamic range supported a good discriminatory power and a satisfactory balance between sensitivity and specificity.

## Background

Bovine tuberculosis, caused by *Mycobacterium bovis *and other closely related mycobacteria of the *Mycobacterium tuberculosis *complex, is endemic in many countries. These mycobacteria can infect a wide range of domestic and wild animals [1-3]. Wild animals become increasingly important in the spread and maintenance of *M. bovis *infection, especially when the efforts to eradicate the disease in livestock have reduced its incidence in domestic cattle [2,4]. The existence of wildlife tuberculosis (TB) reservoirs and the difficulty of controlling the disease in these species is the most important complication in eradication programs [3]. Well known examples of wildlife TB reservoirs include the badger (*Meles meles*) in the United Kingdom and Ireland [5,6], the brushtail possum (*Trichosurus vulpecula*) in New Zealand [3], the white-tailed deer (*Odocoileus virginianus*) in the north of the United States of America [7], the buffalo (*Syncerus caffer*) in South Africa [8,9], or the bison (*Bison bison*) in Canada [10].

In Spain, TB prevalence is relatively low in cattle (0.42 in 2006), but the infection persists in other livestock including goats and free-ranging swine, and there is a wide range of wild animal species susceptible to this disease [11]. Previous research suggested inter-specific transmission of the *M. tuberculosis *complex among wild ungulates and livestock [11-14]. The European wild boar (*Sus scrofa*) is one of the ungulates involved in the epidemiology of tuberculosis in Spain. Recent epidemiological, pathological and microbiological evidence strongly suggests that, at least in Spanish Mediterranean ecosystems, wild boar are able to maintain TB infection in the wild and most likely can transmit the disease to other species, acting as a true wildlife reservoir [15]. Depending on risk factors such as host age and management including feeding and fencing, wild boar TB prevalence ranges based on gross pathology from 18 to 100% [16,17]. The diagnosis of *M. bovis *infection in live animals generally depends on the cellular immune response to *M. bovis *antigens in the first stages of the infection [18]. The most usual technique is the hypersensitivity test, based on the intradermal injection of raw antigens [19-21]. This skin testing technique, described by Robert Koch, is still the most widely used tuberculosis diagnostic method in livestock. It is also used in wild ruminants [22,23]. However, skin tests have a limited sensitivity, and non specific reactions may occur in animals sensitized by mycobacteria other than those of the *M. tuberculosis *complex [24,25].

In wild animals, any diagnostic test has an associated risk during the capture, both for the people who handle the animal and for the animal itself, due to handling stress and injuries. Moreover, preliminary results of skin testing in wild boar of known TB status suggest a low sensitivity (unpublished data). Thus, the possibility of a test based on a single sampling would be highly desirable for assessing the prevalence, studying the mechanisms of transmission and monitoring the effects of control measures.

While the delayed-type hypersensitivity reaction is indicative of infection or exposure, antibody formation appears to be more closely related to the extent of bacterial multiplication and antigenic load in the infected individual. ELISA testing is not routinely used in bovine TB control programs mainly due to a reduced sensitivity [26], although it has been suggested to be used as a complement to the tuberculin test, especially for the detection of anergic tuberculous cattle [27,28].

The aim of this study was to develop and validate an ELISA test for the detection of *Mycobacterium bovis *antibodies in wild boar serum. To achieve this goal, the humoral immune response measured by this test was first measured in captive wild boar sensitized with inactivated bacterial antigens and then results were validated with sera obtained from wild boar of known microbiological TB status.

## Results

### Humoral response to mycobacterial antigens

**Figure 1:**
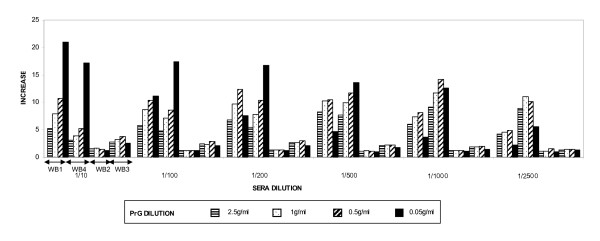
Increase rate (S2/S1) in the optical density reading of the ELISA with bovine PPD and protein G in WB1 and WB4 (immunized with *M. bovis*), WB2 (*M. avium paratuberculosis*) and WB3 (*M. avium*).

The two *M. bovis *immunized wild boars (WB1 and WB4) developed a large increase in the level of antibodies between pre-immunization (S1) and 30 days post-immunization (S2) serum samples while showing a much smaller increase at 90 days post-immunization (Table 1; Figure 1). The other two wild boar, immunized with *M. avium *(WB3) and *M. paratuberculosis *(WB2), showed a much smaller increase in the antibody level against bovine PPD between the pre-immunization sampling and controls S2 and S3 (Table 1).

**Table 1:** Average OD readings for serum dilution and protein G combination.

		**Serum dilution**						**Protein G dilution**			
		***1/10***	***1/100***	***1/200***	***1/500***	***1/1000***	***1/2500***	**2.5 **μg/ml	**1 **μg/ml	**0.5 **μg/ml	**0.05 **μg/ml
**WB1 + WB4**	**S2/S1**	9,2805	9,2610	9,6143	9,5895	9,1172	6,4596	6,3067	8,3368	10,702	11,129
	**S3/S1**	10,465	11,061	11,38	11,574	11,730	9,1666	7,5116	10,252	12,074	13,751
	**S1**	0,1758	0,1089	0,0855	0,0596	0,0465	0,0355	0,1428	0,0981	0,0728	0,0275
	**S2**	1,0053	0,8198	0,7176	0,57	0,4293	0,2521	0,7832	0,729	0,6658	0,3514
	**S3**	1,0706	0,9049	0,7892	0,6605	0,5357	0,3438	0,8763	0,8273	0,7576	0,4086
**WB2**	**S2/S1**	1,4873	1,2624	1,2840	1,1513	1,2033	1,1837	1,2756	1,2966	1,3386	1,1372
	**S3/S1**	5,9384	5,5608	5,0313	3,6979	2,8577	2,3318	4,0252	4,7892	5,1064	3,0244
	**S1**	0,0941	0,0669	0,055	0,0523	0,0414	0,0305	0,094	0,062	0,0491	0,0217
	**S2**	0,1473	0,0841	0,0719	0,061	0,0504	0,0353	0,125	0,0844	0,0657	0,0248
	**S3**	0,5771	0,3818	0,2885	0,2023	0,1224	0,0686	0,4152	0,3324	0,2779	0,0683
**WB2**	**S2/S1**	3,1229	2,4333	2,6135	2,0944	1,8366	1,4173	2,2195	2,3216	2,5731	1,8978
	**S3/S1**	4,0662	3,3376	4,0625	3,1850	2,8014	2,1894	3,0310	3,3525	3,7237	2,9875
	**S1**	0,1119	0,0671	0,054	0,0611	0,0485	0,0383	0,1052	0,0704	0,0543	0,0241
	**S2**	0,3505	0,1645	0,145	0,1318	0,0914	0,0543	0,2504	0,8477	0,1515	0,0464
	**S3**	0,41	0,2215	0,2269	0,1994	0,1374	0,0839	0,3213	0,8577	0,213	0,0731

All protein G concentrations at each serum dilution, and all serum dilutions at each protein G concentration.

WB1+WB4: Sum of wild boar 1 and wild boar 4 results. S2/S1 and S3/S1: Increase rate between pre- and post-immunization results. S1, S2 and S3: OD values.

The optical densities (OD) obtained showed better discrimination at greater dilutions, but at serum dilutions higher than 1/200 we observed a decrease in OD values converging to blank readings. Sera reacted with both conjugates, Protein G and Protein A, but OD values were more homogeneous and the discrimination was higher with Protein G. Finally, serum dilution and conjugate Protein G concentration combinations showing the highest increases between S1 and S2 were retained for validation with the known status sera: 1/10–0.05 μg/ml, 1/200–2.5 μg/ml, 1/200–0.5 μg/ml and 1/200–0.05 μg/ml.

### Known status sera analysis and determination of cut-off value

The summary of ELISA results in Table 2 shows that at the highest semi-sum, specificities were > 93% and sensitivities > 69% in all combinations. The best semi-sum was obtained at 1/200 serum dilution and 2.5 μg/ml conjugate concentration. These dilutions yielded the highest sensitivity (80.26%) among all tested combinations, but at the same time the specificity was reduced to 95.61%. The next best combination was that of a 1/200 serum dilution and a conjugate concentration of 0.05 μg/ml, with 72.60% sensitivity and 96.43% specificity.

**Table 2:** Sensitivity, specificity, semi-sum, specificity discriminating index (SpDI) and sensitivity discriminating index (SeDI) values of different combinations of sera and PrG conjugate.

**ELISA**	**SENSITIVITY**	**SPECIFICITY**	**SEMI-SUM**	**SpDI**	**SeDI**
**1/10–0.05 ug/ml**	70,67%	93,81%	0,8224	11,4076	3,1979
**1/200–2.5 ug/ml**	80,26%	95,61%	0,8794	18,3000	4,8444
**1/200–0.5 ug/ml**	69,33%	97,35%	0,8334	26,1156	3,1743
**1/200–0.05 ug/ml**	72,60%	96,43%	0,8452	20,3288	3,5196

In order to assess the reproducibility of the ELISA and to validate the test, the two latter combinations (1/200 and 2.5 μg/ml, and 1/200 and 0.05 μg/ml) were repeated in two different days. It was observed that the replicates in case of the combination 1/200 and 0.05 μg/ml varied less than those of the other combination. Besides, the coefficient of variation (CV) between days of each test was smaller and the dynamic range was broader too for the 1/200 and 0.05 μg/ml combination (Table 3).

**Table 3:** Descriptive statistics at two selected combinations.

	***COMBINATION 1/200–0.05 μg/ml***		***COMBINATION 1/200–2.5 μg/ml***	
	***ELISA 1***	***ELISA 2***	***ELISA 1***	***ELISA 2***
**n**	182	182	183	183
**Average**	0,3578	0,4152	0,5485	0,4862
**SD**	0,4633	0,5475	0,3855	0,3572
**CV**	129%	132%	70%	73%
**OD max**.	1,3902	1,6046	1,3907	1,1906
**OD min**.	0,0127	0,0213	0,0544	0,0604
**Range**	1,3775	1,5833	1,3363	1,1302
**CV average**	13,85%		19,91%	
**CV max**.	46,74%		94,32%	

**SD**: Standard deviation; **CV**: Coefficient of variation; **OD max**.: Maximum optical density value; **OD min**.: Minimum optical density value; **CV average**: Average of coefficient of variation for each serum two replicates; **CV max**.: Maximum coefficient of variation for each serum two replicates.

**Figure 2:**
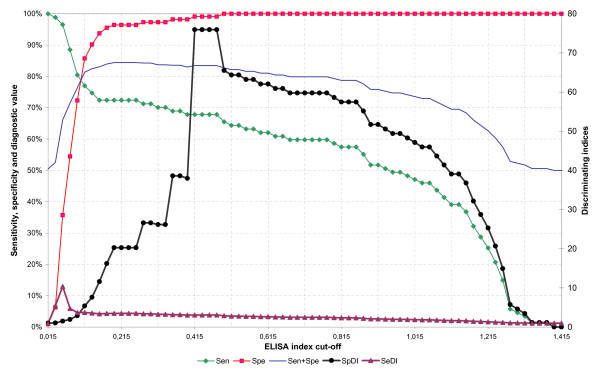
Dynamics of sensitivity (Sen), specificity (Spe), diagnostic value semi-sum (SE+SP), specificity discriminating index (SpDI) and sensibility discriminating index (SeDI) at selected conditions (serum sample dilution 1/200 and PrG conjugate 0.05 μg/ml).

**Figure 3:**
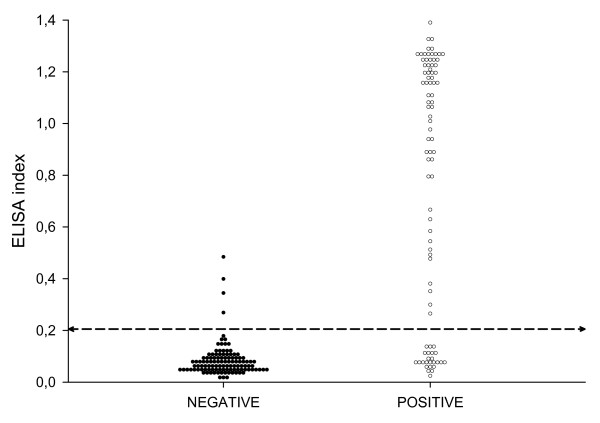
**Histogram of the distribution of the ELISA index values at the defined conditions (1/200 and 0.05 μg/ml) and according to the status of the animals: positive (POSITIVE) or negative (NEGATIVE).** The horizontal line represents the chosen cut-off value.

Figure 2 shows the dynamics of sensitivity, specificity, diagnostic value semi-sum, SeDI and SpDI values for the ELISA with 1/200 and 0.05 μg/ml sera/conjugate combination. It can be seen that the range between 0.115 and 0.655 has diagnostic values over 80%, with a slightly higher primary peak between 0.195 and 0.275 and a secondary one between 0.415 and 0.475. The SeDI and SpDI peak on both sides of the first peak, and indicate the optimal performance when either sensitivity or specificity is the main focus for the test. If a good balance is the goal, then an intermediate value should be the choice. In this case, and taking into account the distribution shown in the histogram (Figure 3) a value at around 0.2 appears to provide the best trade-off. This figure shows that there are at least bimodal distributions both for reference negative and positive sera. This bimodal distribution is less clear for the negative set since all but four sera locate below the 0.2 cut-off. The positives have a highly polarized distribution with markedly high modes at both ends of the EI range and an almost continuous distribution between them. Therefore a cut-off of 0.200 was finally chosen as the one yielding the best balance between sensitivity and specificity and locating to a region where no values were found thus indicating a biological separation between EI populations.

## Discussion

To date, studies are not available on the experimental use of antigens to characterize the immune response of wild boar and pigs against *M. bovis*. Herein, inactivated mycobacterial antigens were successfully used to stimulate a specific humoral immune response in healthy wild boar. Injection of the antigens caused an increase of specific antibodies, but the administration of a second antigen dose did not raise the antibody levels so much. A strong cross reaction between antibodies specific for *M. avium *and *M. avium paratuberculosis *(WB 2 and WB 3) and the bovine PPD ELISA was not observed except to some extent for WB2 after the boosting. Thus, the immunization assay provided negative and positive *M. bovis *control sera to use in the development of the ELISA, as well as for reference in the future.

It is generally recognized that humoral immunity is not important for tuberculosis diagnosis [29], but it may nonetheless be useful to detect animals with severe illness since antibody concentration is related to lesion distribution and severity, as well as to the number of bacilli [18,22,26,30-32]. Mycobacterial infections induce antibody production in ruminants, but the profile of immunoglobulin (Ig) expression in *M. bovis *infected animals is poorly understood [33-36]. In previous studies, Ig heavy and light chains were up-regulated in European wild boar infected with *M. bovis *[37]. However, serum determinations suggested elevated levels of IgG in uninfected wild boar when compared to *M. bovis *infected animals [38]. The mechanism of Ig differential expression in *M. bovis *infected wild boar is unknown but may reflect different stages during mycobacterial infection. Furthermore, differential gene expression analysis in response to mycobacterial infection in wild boar suggest that antibody responses against *M. bovis *may be important in natural infections of wildlife species and may be used for bTB surveillance and treatment monitoring [35,39-41].

Previous studies using ELISA tests achieved 74% sensitivity and 90% specificity in cattle [26], and 79% to 98% specificity and 37% sensitivity in ELISA used in badgers [41-43], although the latter was increased for badgers with progressive tuberculosis [44]. In addition, a positive ELISA result in badgers was correlated with an enhanced likelihood of a future positive culture result [45]. Various mycobacterial antigens have also been used for bovine tuberculosis ELISA in other studies. This includes for example ELISA based on MPB70 and MPB83 in cattle, which have reported specificities of 89% and 96.4%, and sensitivities of 18.1% and 37.5% [46,47]. The results obtained in the current study, thus, indicate that the ELISA performance was substantially better for wild boar, especially in terms of sensitivity (72.60%) and without a significant loss in specificity (96.43%) despite the fact that it is generally recognized that the sensitivities and specificities of ELISA protocols for serodiagnosis of bovine tuberculosis are low as compared to those for other diseases [48].

At the chosen cut-off, there were a few reference negative animals that had medium level antibody reactivity against bovine PPD. It might be possible that some of these animals were infected individuals without visible lesions and bacteria not found, or true non-infected but exposed to *M. bovis *infection, that is, potentially resistant animals. Actually, ELISA positive results are usually considered only as evidence of exposure [17] and not necessarily of current infection. Infections with other mycobacteria causing cross-reactivity cannot be completely ruled out although, given the origin of the animals in infected areas and the EI gap to the rest of negative controls, we think they are less likely to be involved. On the other hand there were animals where *M. bovis *was isolated that had very low EI readings. These animals might represent recently infected individuals not having developed yet a humoral immune response or anergic animals with limited immune responses due to poor body condition.

## Conclusion

We conclude that the use of the serological ELISA test developed herein may contribute to the diagnosis of TB in wild boar and probably also in pigs, with an acceptable sensitivity and specificity and without the need to handle the animals twice as in the skin test. This ELISA test could be used in the control of TB in wild boar through "test & cull" schemes and in large-scale surveys [49]. These results support the use of the ELISA test to complement other techniques based on cellular response to characterize mycobacterial infection in wild boar.

## Methods

### Reference sera

#### Wild boar immunization

In order to obtain serum controls to be used in the ELISA, four captive adult wild boars living in a *M. bovis *free area were used. On day 0, wild boar 1 (WB1) and 4 (WB4) were immunized by the subcutaneous route with 1 ml of a suspension containing 10^3^–10^6 ^colony-forming units (CFU; 2.5 mg) of heat inactivated *M. bovis*. Wild boar 3 (WB3) was immunized with 1 ml (10^3^–10^6 ^CFU) of heat inactivated *M. avium *(*M. bovis *and *M. avium *were kindly provided by CZ Veterinaria), and wild boar 2 (WB2) with 1 ml (2.5 mg) of heat inactivated commercial *M. avium paratuberculosis *vaccine (Gudair^® ^CZ Veterinaria, S.L., Spain). Prior to immunization a serum sample (S1) was taken from each animal. Thirty days later a second serum sample (S2) was taken and all the animals were re-immunized in the same way. Ninety days later a third serum sample (S3) was taken from the four animals. All sera were stored frozen at -20°C until used for testing. All animal use was supervised by the Neiker Committee on Animal Experimentation in accordance with Spanish laws.

#### Wild Boar of known TB status

In order to validate the ELISA, 185 sera obtained from known TB status cases (known status sera) were analyzed. This material included 73 sera from naturally TB positive wild boar, defined as individuals with both tuberculosis compatible lesions [50] and *M. tuberculosis *complex isolation and PCR confirmation (data not shown). The material also included 112 sera from TB negative wild boar, defined as animals with no visible TB-compatible lesions and negative culture. These sera were analyzed with the optimized ELISA protocol, using S3 of WB1 or WB4 as positive control and S1 as negative control.

### Indirect ELISA optimization

Bovine tuberculin purified protein derivative (bovine PPD) (CZ Veterinaria, S.L., Spain) was diluted to 5 μg/ml in carbonate buffer (63 mM, pH 9.6). Plates (High binding ELISA microplate, Greiner bio-one, Germany) were coated with 100 μl of diluted PPD and used in fresh, without any storage. Serum samples and controls, were adsorbed (1/1) with *Mycobacterium phlei *saline suspension (5 gr/l; Dr. O. Fuentes, INIA, Spain), to remove non-specific anti-*Mycobacterium spp*. antibodies. After an overnight incubation at 4°C, samples were diluted in PBS-Tween (0.14 M NaCl; 3 mM KCl; 10 mM Phosphate buffer; 0.05 Tween 20; pH 7.4) and 100 μl of each dilution were added in duplicate in contiguous wells of the bovine PPD coated plate and incubated for 2 hours at room temperature. After three washings with PBS-Tween, 100 μl of Protein A or G were added and incubated for two hours at room temperature. Again after three washings, 100 μl of ABTS substrate (Sigma-Aldrich) were added to each well and incubated for 20 minutes in the dark at room temperature. Optical densities (OD) were read with 405 and 450 nm filters (MultiskanEX, Thermolabsystems). Sample result was expressed as an ELISA index (EI) that was calculated as the ratio of the mean sample OD to the mean OD of the positive controls.

Optimal dilutions of sera were determined by the evaluation of the reactivity of serial dilutions (1/10; 1/100; 1/200; 1/500; 1/1000 and 1/2500) and in combination of serial dilutions of Protein G or Protein A conjugated with recombinant peroxidase from *Streptococcus sp*. (2.5 μg/ml; 1 μg/ml; 0.5 μg/ml; 0.05 μg/ml) (Sigma-Aldrich) with reference serum samples S1, S2 and S3 from the four immunized wild boar analyzed in duplicate. The serum dilution and Protein G concentration at which the ratio between S2 and S1 ODs were higher and apparently more representative were selected for testing the whole set of known status sera. These sera were then tested only at 4 different combinations (serum dilution-PrG concentration): 1/10–0.05 μg/ml, 1/200–2.5 μg/ml, 1/200–0.5 μg/ml and 1/200–0.05 μg/ml.

### Indirect ELISA validation

ELISA indices of the whole set of known status sera were entered in a spreadsheet (RA Juste, unpublished), that allowed to calculate sensitivity and specificity for each cut-off. This spreadsheet also calculates the semi-sum of sensitivity and specificity (diagnostic value ranging from 0 to 100) and the ratios of specificity to sensitivity (specificity discriminating index – SpDI) and of sensitivity to specificity (sensitivity discriminating index – SeDI). The spreadsheet also plots all the values in a single graph that allows seeing at once the behaviour of all these relevant variables along the dynamic range of the test. A verification of these analyses was made using the SigmaPlot (Addlink, Barcelona, Spain) graphics package that was also used to plot the positive and negative known status sera EI histogram and to calculate the 95% sensitivity and specificity confidence intervals at each cut-off.

The final cut-off for the test was chosen as that that yielded the highest semi-sum, was located in a region where small changes in its numerical value did not change substantially the semi-sum (Figure 2), and in the histogram was located in the widest gap without values (Figure 3).

## Authors' contributions

OA and JV performed the field work and collected all the samples of the study. OA carried out the laboratory work and generated the immunological data. OA, MB, RAJ, JV and CG designed the experiment. The immunological analyses were designed by OA, MB and RAJ. MB was the project leader and coordinated and supervised all the study. RAJ participated on data analysis and study supervision. OA wrote the manuscript and JF, CG, JV, MB and RAJ contributed to draft it. All authors read and approved the final manuscript.
